# Supplementary material for: Lineage Conversion in Pediatric B-Cell Precursor Acute Leukemia under Blinatumomab Therapy
Source: Int J Mol Sci. 2022 Apr 5;23(7):4019. doi: 10.3390/ijms23074019 (PMC8999738; doi:10.3390/ijms23074019)
Supplement: Supplementary file 1 [file ijms-23-04019-s001.zip › ijms-1634848-supplementary/ijms-1634848-supplementary.pdf]

Table S1
Clinical and Laboratory Features of Patients at Initial Diagnosis

**Clinical data**	**Pt #1**	**Pt #2**	**Pt #3**	**Pt #4**	**Pt #5**	**Pt #6**
**Age/Sex**	12 y/F	3 y/F	10 y/F	14 d/F	11 mo/M	6 mo/F
**PB examination**						
Total WBC, x10^9/L	670	100	7.6	120	22	70.4
Hemoglobin, g/L	49	N/A	99	85	62	61
Thrombocytes, x10^9/L	43	N/A	297	47	4	86
**BM examination**						
% of leukemic cells	N/A	N/A	12.8	92.6	84.7	86.4
Immunophenotype of leukemic cells (co-expressions)	BI-ALL (N/A)	BI-ALL (N/A)	BI-ALL (CD33, NG2)	BI-ALL (CD15, CD33, NG2)	BIII-ALL (CD33, NG2)	BI-ALL (N/A)
Cytogenetic findings	t(4;11)(q21.3-q22.1;q23.3)	t(12;19)(p13;p13)	t(11;19)(q23.3;p13.3)	t(4;11)(q21.3-q22.1;q23.3)	t(11;19)(q23.3;p13.3)	t(4;11)(q21.3-q22.1;q23.3)
Fusion gene	*KMT2A::AFF1*	*TCF3::ZNF384*	*KMT2A::MLLT1*	*KMT2A::AFF1*	*KMT2A::MLLT1*	*KMT2A::AFF1*

PB – peripheral blood; WBC – white blood cells; BM – bone marrow; pt – patient; y – years; mo – months; d – days; F – female; M – male.

Table S2.

GenBank accession numbers for breakpoint junctions' sequences of the lineage switch patients.

Case	DNA forward breakpoint junction	DNA reciprocal breakpoint junction	RNA breakpoint junction
#1	MN238616	-	MZ962468
#2	OK635613	-	OK017417
#3	MZ962618	MZ962634	MZ962579
#4	OK017418	OK017421	OK017415
#5	Submission ID: 2547330	MZ962630	MZ962527
#6	OK017418	OK017420	OK017415

Table S3.

PCR primers used for breakpoint junctions' evaluation

Case	DNA forward breakpoint junction	DNA reciprocal breakpoint junction	RNA breakpoint junction
#1	5'-GATGGAGTCCACAGGATCAGAGT-3'<br>5'-TTCCTCTGACTTGCCTTAGG-3'	-	5'-AGGAGAATGCAGGCACTTTGA-3'<br>5'-GCGGCCATGAATGGGTC-3'
#2	5'-AGCTTTGCATCAGTGTGGG-3'<br>5'-TTTGTCTATCTGACCACATCCTTCC-3'	-	5'-ACTCAAGCAATAACTTCTCGTCCAG-3'<br>5'-TGTGGGGATAGAAGGCCAGA-3'
#3	5'-TGGTAGTGGGTGCTTGTAATC-3'<br>5'-AGTCTTGTTATGTTTCTGAGTCTGG-3'	5'-AGTCTCACTCTGTCGCCCAGG-3'<br>5'-GCCTTCACATTTGCAACAGATAATAATGC-3'	5'-CGCCTCAGCCACCTACTACAG-3'<br>5'-TGGGCTTCTTGCGCAGTT-3'
#4	5'-ACCTTACAACTGTTTCGTATATTACAG-3'<br>5'-TTGGTGGTAGAGAGACGGGG-3'	5'-TGATATTCTTGTCTTTTACTGTAGGG-3'<br>5'-TGGAAGGGCTCACAACAGACTTGG-3'	5'-AGGAGAATGCAGGCACTTTGA-3'<br>5'-AGGTCGTCTTCGAGCATG-3'
#5	5'-AAGCTAGGTTGAAATCTGAATGTTG-3'<br>5'-AATTTTCTAATCTTGCATAATCCTTGG-3'	5'-GCGTGAGCCACCGCATCTGG-3'<br>5'-GCAAAGCACTGTATTAAGACGGAAAAGAGG-3'	5'-AGGAGAATGCAGGCACTTTGA-3'<br>5'-TGGGCTTCTTGCGCAGTT-3'
#6	5'-CTGAATCCAAACAGGCCACCACT-3'<br>5'-TACAAAGAGGGCCTCTTTTAATTATG-3'	5'-ATAGATCATATAGCTTAGGCCATTTGAAAGG-3'<br>5'-TCAAATGCTGTTTGAGACATCAGTG-3'	5'-GATGCCTTCCAAAGCCTACCTG-3'<br>5'-AGGTCGTCTTCGAGCATGGA-3'

Table S5.

Percentage of cells with respective molecular aberration by FISH, %

Patient	Prior to blinatumomab	After blinatumomab
#1	90%	N/A*
#2	30%	100%
#3	100%	98%
#4	100%	100%
#5	90%	95%
#6	35%	90%

*16% of blasts by MFC

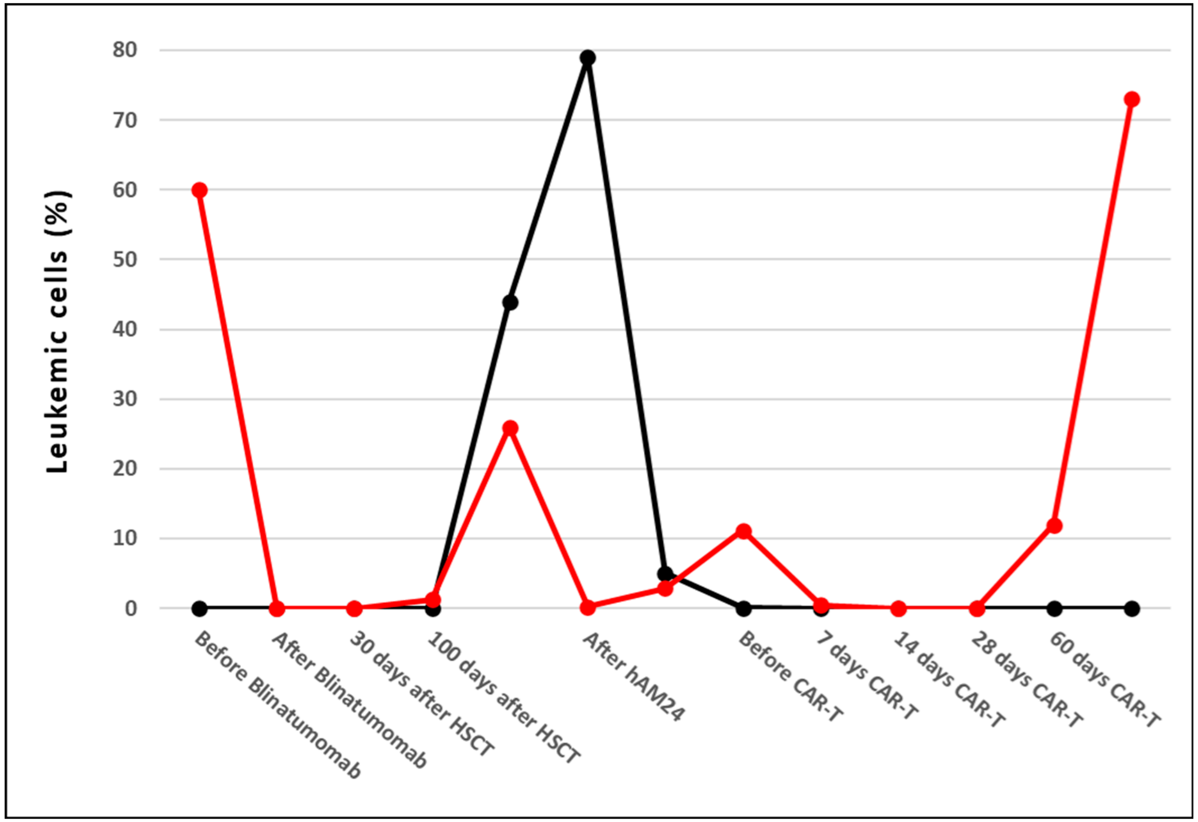

Figure S1. Kinetics of leukemic cells in pt#3. Red line indicates changes in percentage of B-lineage blasts while black line – kinetics of cells with unclassifiable immunophenotype.

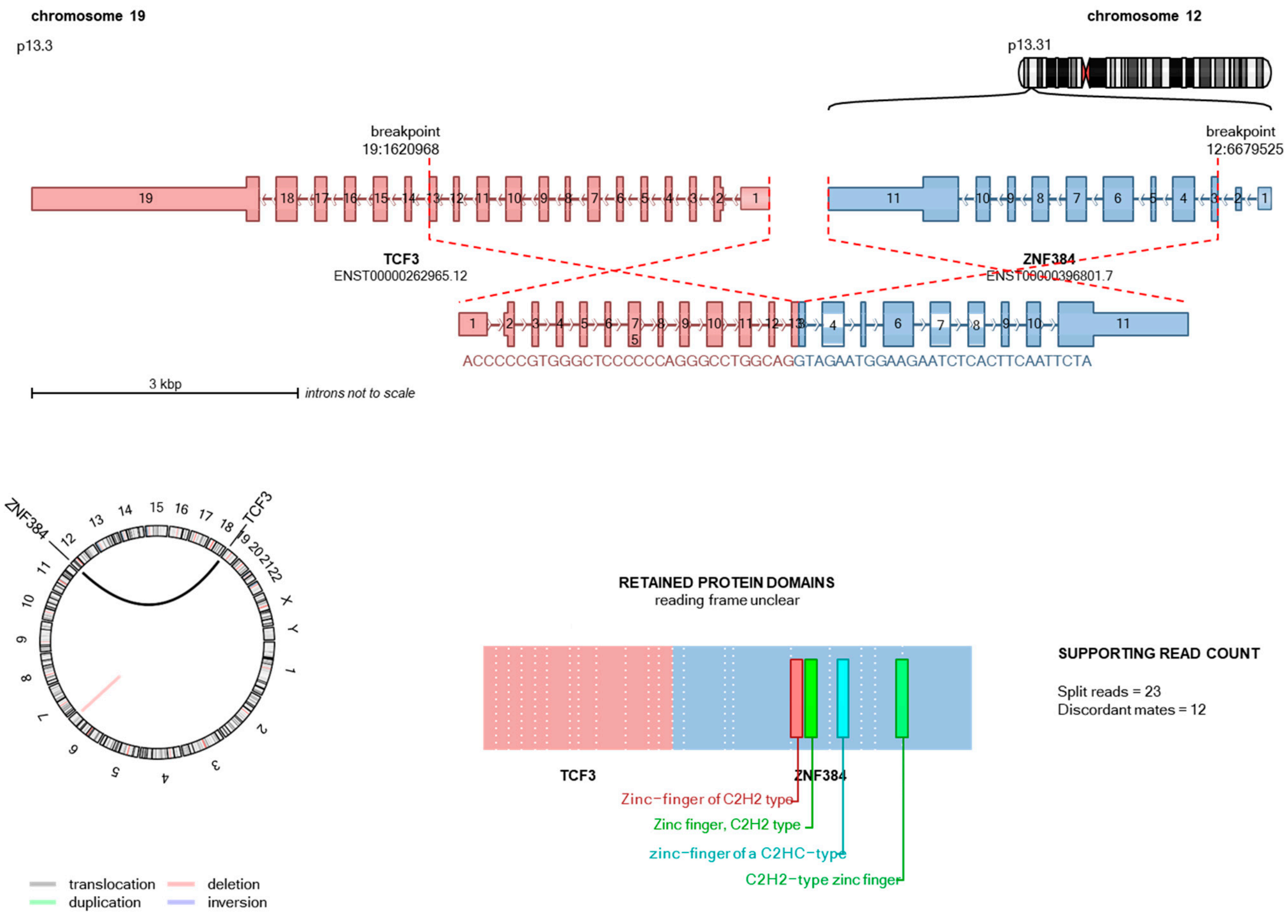

Figure S2. t(12;19)(p13;p13)/*TCF3::ZNF384* fusion transcript in patient #2 – the result of RNAseq study with Arriba algorithm analysis

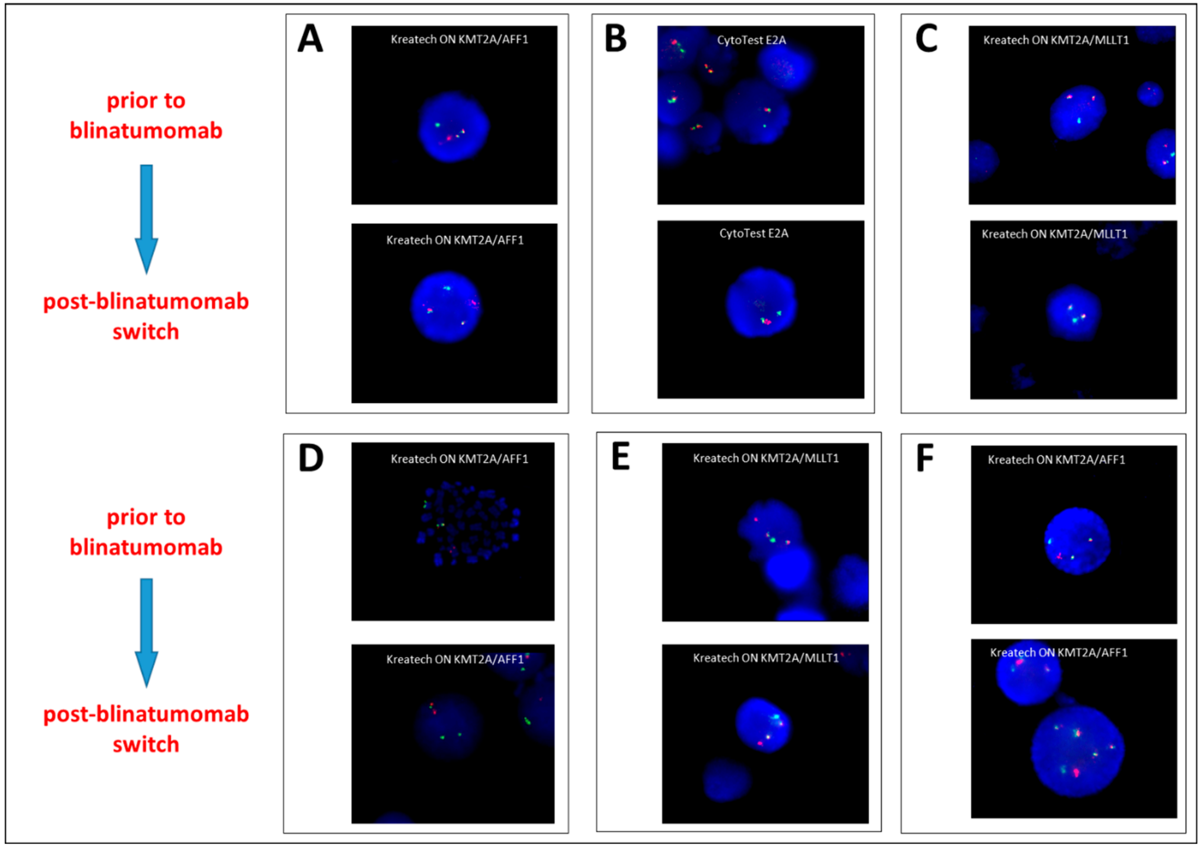

Figure S3. Conventional cytogenetics and FISH in lineage switch patients demonstrating the consistency of the main chromosomal translocations. **A** – t(4;11)(q21.3-q22.1;q23.3)/*KMT2A::AFF1* in pt#1; **B** – *TCF3* gene rearrangement within t(12;19)(p13;p13)/*TCF3::ZNF384* in pt#2; **C** – t(11;19)(q23.3;p13.3)/*KMT2A::MLLT1* in pt#3; **D** – *KMT2A* gene rearrangement within t(4;11)(q21.3-q22.1;q23.3)/*KMT2A::AFF1* in pt#4; **E** – t(11;19)(q23.3;p13.3)/*KMT2A::MLLT1* in pt#5; **F** – t(4;11)(q21.3-q22.1;q23.3)/*KMT2A::AFF1* fusion gene in pt#6

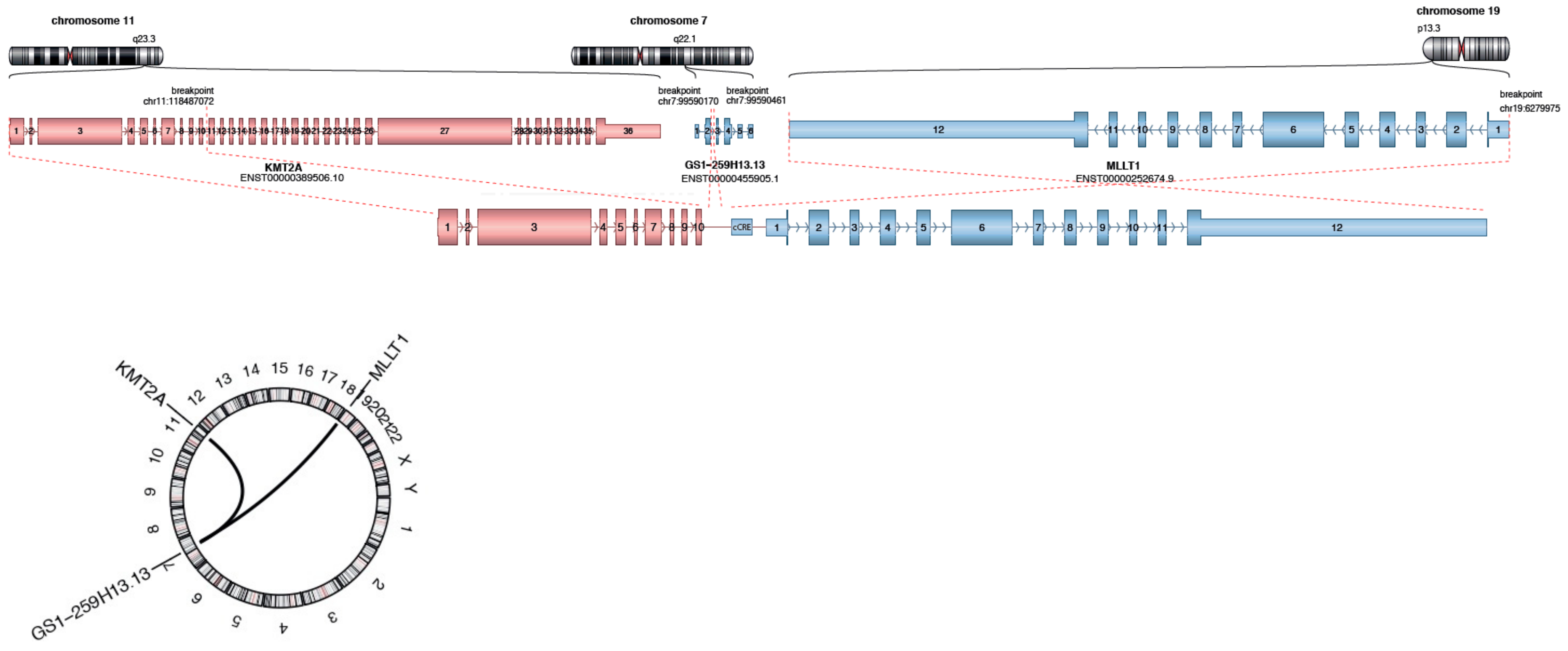

Figure S4. Complex *KMT2A*::7q22.1::*MLLT1* upstream DNA fusion gene in patient #5 – the result of *KMT2A*-targeted panel and Sanger sequencing study illustrated by Arriba algorithm.

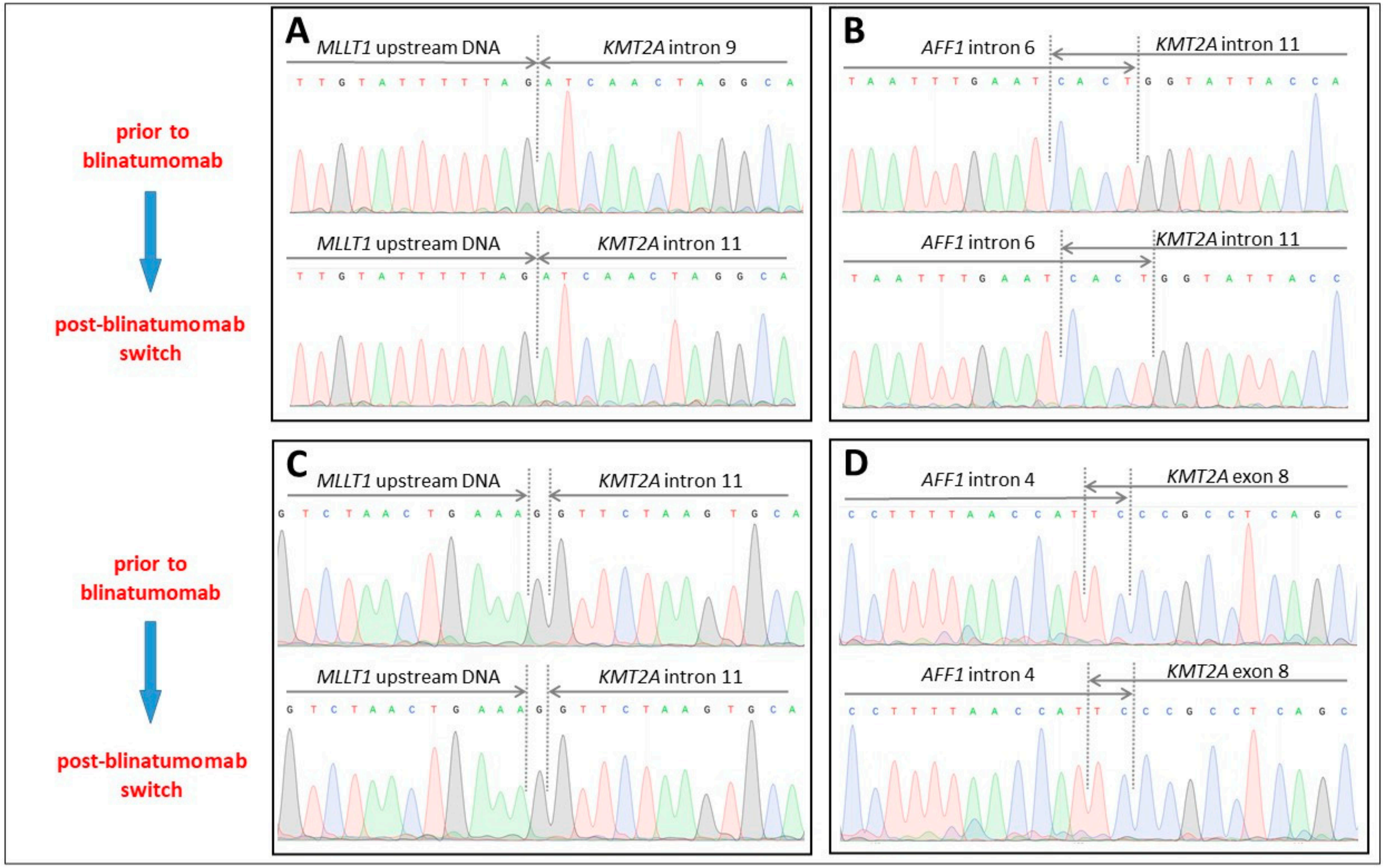

Figure S5. Molecular studies in lineage switch patients demonstrating the consistency of the main chromosomal translocations. **A** – *MLLT1::KMT2A* reciprocal fusion gene in pt #3. **B** – *AFF1::KMT2A* reciprocal fusion gene in pt #4. **C** – *MLLT1::KMT2A* reciprocal fusion gene in pt #5. **D** – *AFF1::KMT2A* reciprocal fusion gene in pt #6.

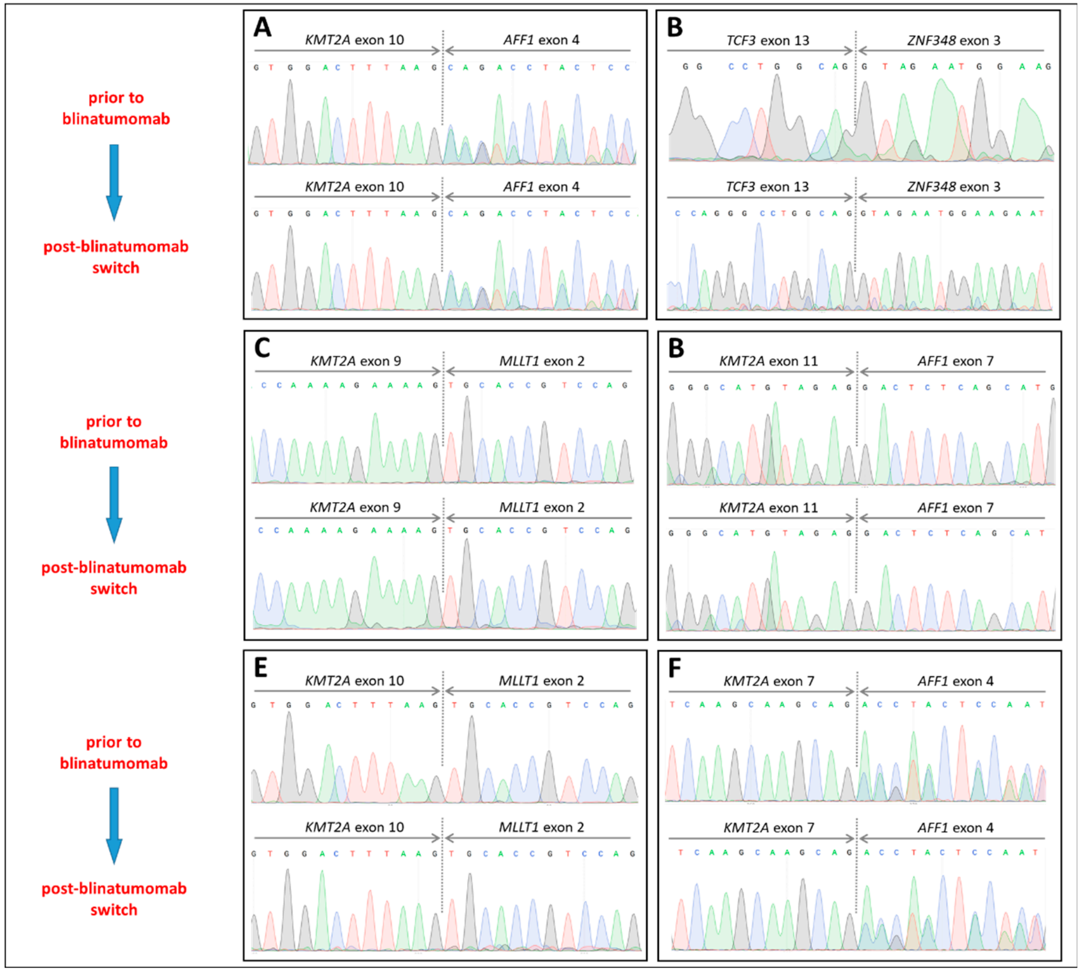

Figure S6. Molecular studies in lineage switch patients demonstrating the consistency of the main chromosomal translocations. **A** – *KMT2A::AFF1* fusion transcript in pt #1; **B** – *TCF3::ZNF384* fusion transcript in pt #2; **C** – *KMT2A::MLLT1* fusion transcript in pt #3; **D** – *KMT2A::AFF1* fusion transcript in pt #4; **E** – *KMT2A::MLLT1* fusion transcript in pt #5; **F** – *KMT2A::AFF1* fusion transcript in pt #6.
